# *Ninjinyoeito* ameliorated cigarette smoke extract-induced apoptosis and inflammation through JNK signaling inhibition in human lung fibroblasts

**DOI:** 10.1186/s12906-022-03574-5

**Published:** 2022-03-31

**Authors:** Kenta Murata, Nina Fujita, Ryuji Takahashi

**Affiliations:** Kampo Research Laboratories, Kracie Pharma, Ltd., 3-1 Kanebo-machi, Takaoka-City, Toyama 933-0856 Japan

**Keywords:** *Ninjinyoeito*, JNK, Apoptosis, Cigarette smoke

## Abstract

**Background:**

Cigarette smoke is a major risk factor for various lung diseases, such as chronic obstructive pulmonary disease (COPD). *Ninjinyoeito* (NYT), a traditional Chinese medicine, has been prescribed for patients with post-illness or post-operative weakness, fatigue, loss of appetite, rash, cold limbs, and anemia. In addition to its traditional use, NYT has been prescribed for treating frailty in gastrointestinal, respiratory, and urinary functions. Further, NYT treatment can ameliorate cigarette smoke-induced lung injury, which is a destructive index in mice; however, the detailed underlying mechanism remains unknown. The purpose of this study was to investigate whether NYT ameliorates cigarette smoke-induced cell injury and inflammation in human lung fibroblasts and determine its mechanism of action.

**Methods:**

We prepared a cigarette smoke extract (CSE) from commercially available cigarettes to induce cell injury and inflammation in the human lung fibroblast cell line HFL1. The cells were pretreated with NYT for 24 h prior to CSE exposure. Cytotoxicity and cell viability were measured by lactate dehydrogenase (LDH) cytotoxicity assay and cell counting kit (CCK)-8. IL-8 level in the cell culture medium was measured by performing Enzyme-Linked Immuno Sorbent Assay (ELISA). To clarify the mechanisms of NYT, we used CellROX Green Reagent for reactive oxygen species (ROS) production and western blotting analysis for cell signaling.

**Results:**

Exposure of HFL1 cells to CSE for 24 h induced apoptosis and interleukin (IL)-8 release. Pretreatment with NYT inhibited apoptosis and IL-8 release. Furthermore, CSE exposure for 24 h increased the production of ROS and phosphorylation levels of p38 and JNK. Pretreatment with NYT only inhibited CSE-induced JNK phosphorylation, and not ROS production and p38 phosphorylation. These results suggest that NYT acts as a JNK-specific inhibitor.

**Conclusion:**

NYT treatment ameliorated CSE-induced apoptosis and inflammation by inhibiting the JNK signaling pathway. Finally, these results suggest that NYT may be a promising therapeutic agent for patients with COPD.

**Supplementary Information:**

The online version contains supplementary material available at 10.1186/s12906-022-03574-5.

## Introduction

Cigarette smoke contains more than 7,000 chemicals, such as oxidative gases and heavy metals. It is well known that cigarette smoke is a major risk factor for various lung diseases, such as chronic obstructive pulmonary disease (COPD). Epidemiological and clinical studies have shown that smokers are significantly more likely to develop emphysema than nonsmokers, and the severity of the disease is directly correlated with the amount of cigarette smoke inhaled [1, 2]. Cigarette smoke causes direct oxidative stress damage on both cellular and extracellular structural elements of the lung, and initiates inflammatory processes that can cause indirect damage to the lungs. Cigarette smoke exposure to human lung fibroblasts has been reported to have various effects, such as apoptosis, cytokine release, and oxidative stress [3, 4]. In particular, oxidative stress plays a central role in initiating and driving the mitogen-activated protein kinase (MAPK) signaling pathway, leading to various cellular responses following cigarette smoke exposure.

*Ninjinyoeito* (NYT), a traditional Chinese medicine consisting of 12 herbs, has been prescribed for patients with post-illness or post-operative weakness, fatigue, loss of appetite, cold limbs, and anemia. In addition to its traditional use, NYT has been recently prescribed for treating frailty in gastrointestinal, respiratory, and urinary functions [5]. In this context, it has been reported that NYT treatment for older patients with COPD can improve the COPD assessment test score, which is designed to measure the impact of COPD on a person’s life and how it changes over time [6]. In addition to the COPD assessment test score, NYT treatment also improves physical symptoms, such as body weight and muscle mass reduction, as well as psychological symptoms, such as depression and anxiety [6, 7]. In an in vivo study, NYT could ameliorate cigarette smoke-induced alveolar damage, as indicated by the destructive index in a mouse model of cigarette smoke-induced COPD. Although this previous report did not investigate the mechanism of action of NYT on lung damage, the authors suggested that NYT has some favorable effects on localized damage to the alveolar wall, including that on fibroblasts [8]. In addition, NYT has also been reported to have radical scavenging activity in hepatocytes [9] and can ameliorate nitric oxide-mediated lung injury in a murine cytomegalovirus (MCMV)-infected model [10]. Therefore, we hypothesized that NYT possibly exerts a therapeutic effect against cigarette smoke-induced cell injury through its anti-inflammatory and anti-oxidant effects.

Thus, the purpose of this study was to clarify whether NYT ameliorated cigarette smoke-induced cytotoxicity and inflammation in human lung fibroblasts and to determine its mechanism of action.

## Materials and methods

### Cell culture and treatment

Human male fetal lung fibroblast cells (HFL1) were purchased from the American Type Culture Collection (ATCC, Manassas, VA, USA). The cells were grown in Ham’s F-12 K medium (Thermo Fisher Scientific Ltd., Waltham, MA, USA) supplemented with 10% fetal bovine serum (FBS; Thermo Fisher Scientific Ltd.) and 1% antibiotics-antimycotics (Thermo Fisher Scientific Ltd.) in a humidified incubator at 37 °C with 5% CO_2_ as recommended by ATCC. Fibroblasts between 5 and 10 passages were used in all the experiments.

### Cigarette smoke extract preparation

Cigarette smoke extract (CSE) was prepared by bubbling smoke from two cigarettes (1.2 mg nicotine and 14 mg tar) without a filter into 20 mL of serum-free Ham’s F-12 K medium at a rate of 1 cigarette/min as previously described [11]. The pH of the medium was adjusted to 7.4 using HCl, and the medium was sterile filtered with a 0.22 μm filter. The preparation was considered to be a 100% CSE concentration. CSE was then used in the subsequent experiments within 30 min of its preparation.

### Plant materials and extract preparation

NYT is composed of 12 dried medicinal herbs described in Table 1, and was supplied by Kracie Pharma Ltd. (Tokyo, Japan) as a dried extract powder. Each plant material was identified by external morphology and authenticated by marker compounds of the plant specimens according to the method of the Japanese Pharmacopeia and the standards of Kracie Pharma Ltd. The NYT extract used in the study was industrially produced in Kracie Pharma Ltd according to the method of the Japanese Pharmacopeia and the standards of Kracie Pharma Ltd. The yield of each NYT extract was within 28.5–34.5%. The purchasing manager purchased each plant material from various companies in China and the employee in Kracie Pharma Ltd. identified by comparing it with the standard specimens we own. Standard specimens are deposited in our laboratory, but we cannot deposit them in a publicly available herbarium as they are considered confidential material. NYT extract (lot No. 15112017) was suspended in serum-free Ham’s F-12 K medium by sonication, and the medium was sterile filtered with a 0.22 μm filter immediately before use. Also, the datasets used and/or analyzed during the current study are available from the corresponding author on reasonable request. The fingerprints of NYT was also performed as previously described [12].

**Table 1:** Medical herb composition of a daily dose of *Ninjinyoeito*

English name	Latin Name	Botanical name	Weight (g)
Rehmannia Root	*Rehmanniae Radix*	*Rehmannia glutinosa (Gaertn.) Libosch. ex Fisch. et C.A. Mey*	4
Japanese Angelica Root	*Angelicae Acutilobae Radix*	*Angelica acutiloba (Siebold et Zucc.) Kitag*	4
Atractylodes Rhizome	*Atractylodis Rhizoma*	*Atractylodes japonica Koidz. ex Kitam*	4
Poria Sclerotium	*Poria*	*Wolfiporia cocos Ryvarden et Gilbertson*	4
Ginseng	*Ginseng Radix*	*Panax ginseng C.A.Mey*	3
Cinnamon Bark	*Cinnamomi Cortex*	*Cinnamomum cassia (L.) J.Presl*	2.5
Polygala Root	*Polygalae Radix*	*Polygala tenuifolia Willd*	2
Peony Root	*Paeoniae Radix*	*Paeonia lactiflora Pall*	2
Citrus Unshiu Peel	*Citri Unshiu Pericarpium*	*Citrus unshiu Markowicz*	2
Astragalus Root	*Astragali Radix*	*Astragalus membranaceus (Fisch.) Bunge*	1.5
Glycyrrhiza	*Glycyrrhizae Radix*	*Glycyrrhiza uralensis Fisch*	1
Schisandra Fruit	*Schisandrae Fructus*	*Schisandra chinensis (Turcz.) Baill*	1

### Cytotoxicity assay

Cytotoxicity and cell viability were measured by lactate dehydrogenase (LDH) cytotoxicity assay (TaKaRa, Shiga, Japan) and cell counting kit (CCK)-8 (Dojindo Laboratories, Kumamoto, Japan), respectively, by following the manufacturer’s instructions. The cells were treated with 2% Triton X-100 as a positive control for the LDH cytotoxicity assay. The cells were then seeded at a density of 1 × 10^4^ cells/well in a 96-well cell culture plate in 10% FBS Ham’s F-12 K medium. After 24 h of incubation, the cells were treated with different concentrations of NYT in 1% FBS Ham’s F-12 K medium for 24 h, and then 5% CSE with NYT was added. After 24 h of incubation, the cell culture plates were centrifuged at 250 g for 10 min, and the supernatant was collected for performing the LDH assay. The LDH cytotoxicity assay kit was used to examine the level of LDH release, and the optical density of the samples was measured using a microplate reader Synergy H1 (BioTek, Tokyo, Japan) at 490 nm. CCK-8 was used to examine the cell viability, and the optical density was measured using a microplate reader set at 450 nm. To investigate the type of cell death that was induced by CSE exposure, the cells were treated with Z-VAD-FMK (Promega, Madison, WI, USA) or necrostatin-1 (Sellmeck Chemical, Houston, TX, USA) 2 h before the CSE exposure.

### Measurement of reactive oxygen species production

The cells were seeded at a density of 1 × 10^4^ cells/well in a 96-well cell culture plate in 10% FBS Ham’s F-12 K medium. After 24 h of incubation, the cells were treated with different concentrations of NYT in 1% FBS Ham’s F-12 K medium for 24 h, and then 5% CSE with NYT was added. After 8 h or 24 h of incubation, CellROX Green Reagent (Thermo Fisher Scientific Ltd.) was added to each well at a concentration of 10 μmol/L for 30 min. The fluorescence of CellROX was measured using the microplate reader Synergy H1.

### Western blotting analysis

The cells were seeded at a density of 2 × 10^5^ cells/well in a 6-well cell culture plate in 10% FBS Ham’s F-12 K medium. After 24 h of incubation, the cells were treated with 100 μg/mL NYT in 1% FBS Ham’s F-12 K medium for 24 h, and then 5% CSE with NYT was added. After 24 h of incubation, the supernatants were collected for an Enzyme-linked Immuno Sorbent Assay (ELISA), and the cells were collected using RIPA buffer (Fujifilm, Tokyo, Japan) supplemented with a protease-inhibitor cocktail (Nacalai Tesque, Kyoto, Japan) and a phosphatase-inhibitor cocktail (Nacalai Tesque). Western blot analysis was performed as previously described [13]. Lysates were centrifuged at 15,000 g for 20 min at 4 °C, and then 10 μg aliquots of protein were separated on 10%–20% SDS polyacrylamide gels, transferred onto polyvinylidene difluoride membranes (Merck Millipore, Burlington, MA, USA), and immunoblotted with the following primary antibodies at 4 °C overnight: rabbit anti-cleaved caspase-3 monoclonal antibody (1:1000, Cell Signaling Technology [CST], Danvers, MA, USA), rabbit anti-cleaved PARP monoclonal antibody (1:1000,CST), rabbit anti-phospho-p44/42 MAPK (Erk1/2) monoclonal antibody (1:1000,CST), rabbit anti-phospho-p38 MAPK monoclonal antibody (1:1000,CST), rabbit anti-phospho-SAPK/JNK monoclonal antibody (1:1000,CST), rabbit anti-phospho-SEK1/MKK4 monoclonal antibody (1:1000,CST), rabbit anti- Erk1/2 monoclonal antibody (1:1000,CST), rabbit anti-p38 MAPK monoclonal antibody (1:1000,CST), rabbit anti-SAPK/JNK monoclonal antibody (1:1000,CST), rabbit anti-SEK1/MKK4 monoclonal antibody (1:1000,CST), and mouse anti-β-actin monoclonal antibody (1:1000; CST). The secondary antibodies used were HRP-conjugated goat anti-rabbit IgG (1:2000; CST) or goat anti-mouse IgG (1:5000; CST). Immunoreactive bands were visualized using an Amersham Imager 680 analyzer (GE Healthcare, Chicago, IL, USA). The band intensity was measured using Fiji software. Since the membranes were cut prior to hybridization with antibodies, we could not provide images of adequate length.

### IL-8 quantification in the medium by ELISA

IL-8 level in the cell culture medium was measured by performing ELISA with human IL-8/CXCL8 (R&D Systems, Minneapolis, MN, USA) by following the instructions in the product manual. Briefly, the collected cell culture medium was centrifuged at 250 g for 10 min, and the supernatant was used as a sample.

### Statistical analysis

All statistical analyses were performed using EZR (Saitama Medical Center, Jichi Medical University, Saitama, Japan), a graphical user interface for R (The R Foundation for Statistical Computing, Vienna, Austria). EZR is a modified version of R commander designed to add statistical functions frequently used in biostatistics. Statistical comparisons were performed using one-way analysis of variance followed by Tukey’s test when comparing three or more groups, or Student’s *t*-test when comparing two groups. Differences were considered significant at *p* < 0.05. All experiments were performed more than three times to confirm the reproducibility.

## Results

### NYT inhibited CSE-induced cytotoxicity and IL-8 release

**Fig. 1:**
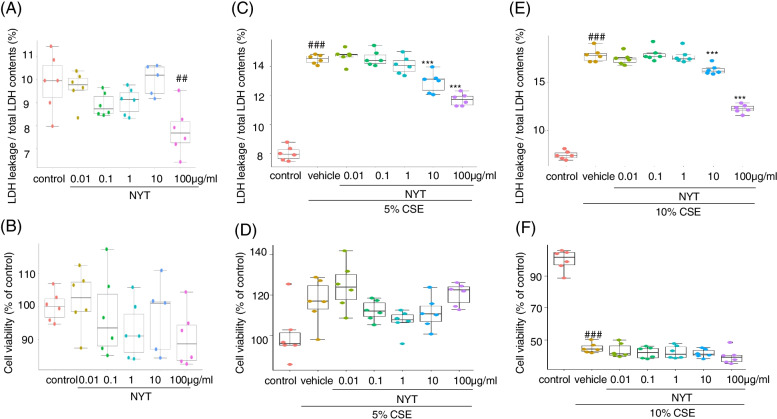
NYT ameliorated CSE-induced LDH leakage. The effect of NYT on cytotoxicity was evaluated using an LDH assay kit or a CCK-8 assay kit without CSE exposure. The cells were treated with NYT for twenty-four hours, and cytotoxicity was measured by LDH leakage (**A**) and cell viability by the CCK-8 assay (**B**). The effect of NYT on cytotoxicity was evaluated using an LDH assay kit or a CCK-8 assay kit with CSE exposure. The cells were treated with NYT for twenty-four hours prior to exposure to 5% (**C**, **D**) or 10% CSE (**E**, **F**). Twenty-four hours after CSE exposure, cytotoxicity was measured by LDH leakage (**C**, **E**) and cell viability by the CCK-8 assay (**D**, **F**). ^##^*p* < 0.01, ^###^*p* < 0.001 vs. control group; ^***^p < 0.001 vs. vehicle-treatment group; Tukey’s test; *n* = 6. CSE: cigarette smoke extract; NYT: *Ninjinyoeito*

We first investigated the effect of NYT on both LDH leakage and CCK-8 cell viability in HFL-1. The cells were treated with different concentrations of NYT for 24 h, and LDH leakage in the medium and cell viability were evaluated. According to our results, NYT treatment inhibited LDH leakage in the medium, but did not elicit any change in cell viability (Fig. 1A, B).

**Fig. 2:**
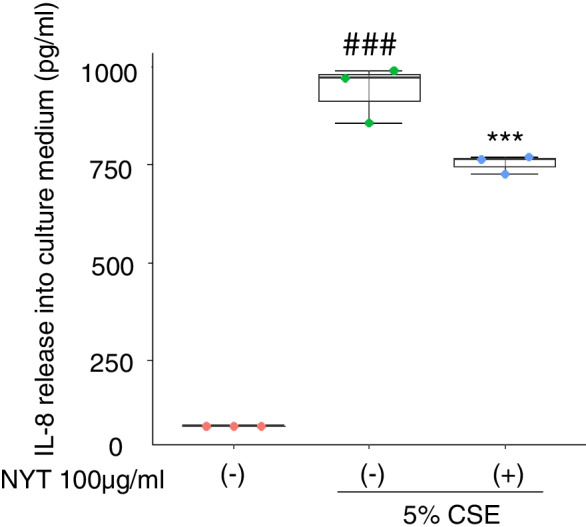
NYT ameliorated CSE-induced IL-8 release. The effect of NYT on CSE-induced IL-8 release was evaluated. The amount of IL-8 in the cell culture medium at twenty-four hours after exposure to 5% CSE was measured using an IL-8 ELISA assay kit. ^###^*p* < 0.001 vs. control group; ^***^p < 0.001 vs. vehicle-treatment group; Tukey’s test; *n* = 3. CSE: cigarette smoke extract; NYT: *Ninjinyoeito*

We further investigated whether NYT treatment could inhibit CSE-induced cytotoxicity. The cells were treated with different concentrations of NYT for 24 h and then treated with 5% or 10% CSE preparation with NYT for 24 h. We found that treatment with both CSE concentrations showed a significant increase in LDH leakage in the medium than that in the control group; however, NYT pretreatment inhibited CSE-induced LDH leakage at both CSE concentrations in a dose-dependent manner (IC50 > 100 μg/ml NYT in 5% CSE, IC50 = 64.2 μg/ml NYT in 10% CSE) (Fig. 1C, E). In the CCK-8 cell viability assay, 5% CSE exposure enhanced and 10% CSE exposure significantly inhibited cell viability at 24 h after exposure, compared with that in the control group. In addition, NYT pretreatment did not alter the CSE-induced changes in cell viability (Fig. 1D, F). In the subsequent experiments, we focused on the effect of NYT against 5% CSE-induced damage in HFL1 cells because 10% CSE had stripped most of cells from the culture dish and we were unable to collect an adequate volume of cells for the subsequent experiments. Next, we investigated whether NYT treatment inhibited CSE-induced inflammation. We evaluated the content of IL-8 in the medium after CSE exposure. CSE exposure significantly increased IL-8 release into the medium 24 h after the exposure than that in the control group; however, NYT pretreatment significantly inhibited this release (Fig. 2). These results suggest that NYT treatment inhibited CSE-induced cell damage and inflammation.

### NYT inhibited caspase-dependent apoptosis signal

**Fig. 3:**
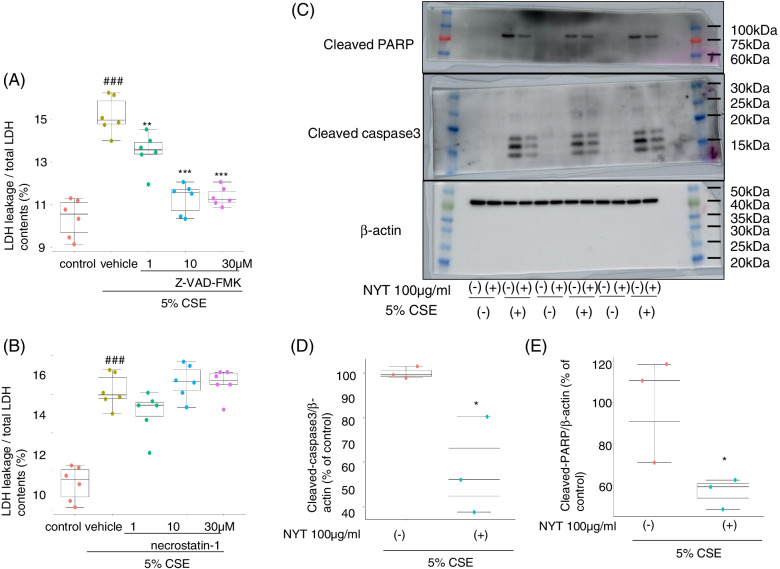
NYT inhibited CSE-induced caspase-dependent apoptosis. The effects of specific inhibitors of apoptosis and necrosis were evaluated using an LDH assay kit. Two hours before exposure to 5% CSE, the cells were treated with either Z-VAD-FMK (**A**) or necrostatin-1 (**B**) to inhibit apoptosis and necrosis, respectively. The cytotoxicity of LDH leakage was evaluated 24 h after CSE exposure. ^###^*p* < 0.001 vs. control group; ^**^p < 0.01, ^***^p < 0.001 vs. vehicle-treatment group; Tukey’s *t*-test; *n* = 6. The effect of NYT on the expression levels of cleaved caspase 3 and PARP was evaluated by Western blotting analysis (**C**-**E**). Twenty-four hours after CSE exposure, the cells were collected, and western blotting analysis was performed to evaluate the expression levels of cleaved caspase 3 (**D**) and cleaved PARP (**E**). The full-length blot images were presented in Supplementary Fig. 1. ^*^p < 0.05 vs. vehicle-treatment group; Student’s *t*-test; *n* = 3. CSE: cigarette smoke extract; NYT: *Ninjinyoeito*

Next, we investigated the type of cell death that occurred in HFL1 cells exposed to 5% CSE. Two hours before CSE exposure, the HFL1 cells were treated with either Z-VAD-FMK or necrostatin-1 to inhibit apoptosis or necrosis, respectively. We observed that only Z-VAD-FMK treatment completely inhibited CSE-induced LDH leakage (Fig. 3A-B). These results implied that CSE exposure to HFL1 cells mainly induced caspase-dependent apoptosis, and that NYT treatment might inhibit CSE-induced apoptosis. Since only Z-VAD-FMK treatment inhibited the CSE-induced LDH leakage, we evaluated the effects on cleaved-caspase 3 and cleaved PARP expression levels at 24 h after CSE treatment as a marker of apoptosis. CSE exposure increased the expression of both cleaved caspase 3 and cleaved PARP, and 100 μg/mL NYT treatment significantly inhibited the increase in the expression of both the proteins (*p* = 0.026 or 0.047, respectively) (Fig. 3C-E). These results indicate that NYT treatment inhibited caspase-dependent apoptosis induced by CSE exposure.

### NYT did not inhibit CSE-induced reactive oxygen species production

**Fig. 4:**
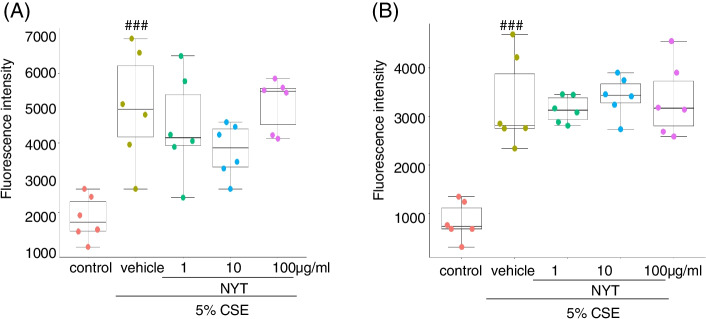
NYT did not change the CSE-induced ROS production. The effect of NYT on CSE-induced ROS production was also evaluated. Intracellular ROS generation was measured using CellROX green reagent at eight (**A**) or twenty-four hours (**B**) after CSE exposure. ^###^*p* < 0.001 vs. control group; Tukey’s test. CSE: cigarette smoke extract; NYT: *Ninjinyoeito*

To clarify the mechanism of action of NYT on CSE-induced apoptosis, we investigated the effect of NYT on reactive oxygen species (ROS) production after CSE exposure. In a previous report, NYT treatment had shown a radical scavenging activity in rat hepatocytes and serum [9, 14]. To measure the ROS production in living cells, the cells were treated with CellROX Green reagent for 30 min at 8 h or 24 h after the CSE treatment. Compared with the control group, the group with CSE exposure showed a significant increase in the ROS production; however, NYT treatment did not change the level of ROS production at either time point (Fig. 4). These results indicated that the anti-oxidant activity of NYT was not involved in the anti-apoptotic and anti-inflammatory effects of NYT.

### NYT inhibited CSE-induced JNK1/2 phosphorylation

**Fig. 5:**
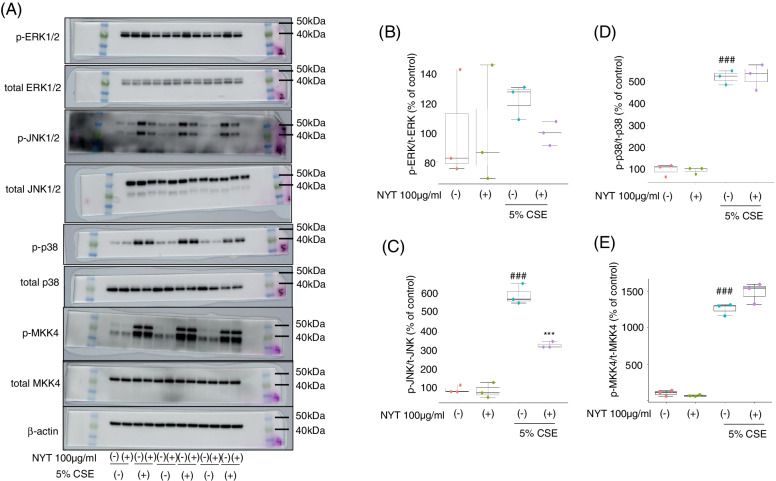
NYT inhibited CSE-induced JNK signal activation. The effect of NYT on the MAPK signaling pathway was evaluated (**A**). Twenty-four hours after CSE exposure, the cells were collected, and western blotting analysis was performed to evaluate the phosphorylation levels of ERK1/2 (**B**), JNK (**C**), p38 (**D**), and MKK4 (**E**). The full-length blot images were presented in Supplementary Fig. 2. ^###^*p* < 0.001 vs. control group; ^***^p < 0.001 vs. vehicle-treatment group; Tukey’s test; *n* = 3. CSE: cigarette smoke extract; NYT: *Ninjinyoeito*

Next, we evaluated the MAPK signal activity after CSE treatment. There are three well-defined subgroups of MAPKs: extracellular signal-regulated kinases (ERK1/2), c-Jun *N*-terminal kinases (JNK), and p38 MAPKs. Without CSE exposure, NYT had no effect on the phosphorylation levels of p38, ERK1/2, and JNK compared with that in the control group. In contrast, 5% CSE exposure significantly increased the phosphorylation levels of p38 and JNK, but not of ERK1/2, at 24 h after the exposure compared with that in the control group. NYT pretreatment inhibited the phosphorylation of only JNK, and not of p38 (Fig. 5A-D). JNKs are phosphorylated by the upstream dual specificity MAP2K, MKK4, or MKK7 molecules. CSE exposure significantly increased the phosphorylation level of MKK4; however, NYT treatment did not change the phosphorylation level (Fig. 5E). These results suggest that NYT acts as a JNK-specific inhibitor.

## Discussion

Here, we investigated whether NYT can ameliorate CSE-induced apoptosis and inflammation in the human lung fibroblast cell line HFL1 and further determined its mechanism of action. We observed that NYT inhibited caspase-dependent apoptosis and IL-8 release induced by CSE exposure. We also showed that NYT specifically inhibited the phosphorylation of JNK induced by CSE exposure. To our knowledge, this is the first study to reveal how NYT treatment ameliorates CSE-triggered apoptosis and inflammation in human lung fibroblasts.

NYT reportedly ameliorates cigarette smoke-induced alveolar damage as indicated by the destructive index; however, it does not affect the air space enlargement, as indicated by the mean linear intercept in a cigarette smoke-induced COPD mice model [8]. This report suggests that NYT has some favorable effects on the localized damage to the alveolar wall, including that on fibroblasts, and that NYT has no effects on other connective structures, such as elastic fibers [8]. However, this report did not investigate the type of cells and the mechanism by which NYT treatment ameliorates cigarette smoke-induced lung injury. While cigarette smoke first targets the epithelium, which acts as a mechanical barrier and contributes to the development of the inflammatory response, water-soluble components of cigarette smoke can pass through the basement membrane and directly interact with the fibroblasts, which play a crucial role in maintaining the integrity of the alveolar structure [15, 16]. In the present study, we demonstrated that NYT treatment inhibited CSE-induced LDH leakage and IL-8 release. These results suggest that NYT has a protective effect against cigarette smoke-induced lung injury in the lung fibroblasts. Additionally, we showed that 5% CSE exposure enhanced and 10% CSE inhibited cell viability in the CCK-8 assay. Another report also showed that 4–6% CSE enhanced and 8–10% CSE inhibited cell viability in an MTT assay [3]. The MTT assay is affected by both the number of living cells and the activity level of mitochondria in the cell. The previous study also demonstrated that 5% CSE exposure decreased cell size, but not mitochondrial membrane potentiation, and that 10% CSE exposure decreased both cell size and mitochondrial membrane potentiation. In the present study, 5% CSE exposure decreased the cell size and did not inhibit cell proliferation; consequently, 5% CSE exposure increased cell density in the culture dish compared to control group.

Oxidative stress plays a central role in initiating and driving signaling pathways that lead to cellular responses following CSE exposure. NYT suppresses the MCMV infection-induced increase in iNOS and NO levels in serum [10]. Therefore, we hypothesized that NYT possibly suppresses CSE-induced apoptosis and inflammation via its anti-oxidant activity; however, NYT treatment did not change the CSE-induced ROS production at any time point. While NYT reportedly inhibits *tert*-butyl hydroperoxide-induced damage via its radical scavenging activity in rat hepatocytes [9], the present results indicate that NYT has no radical scavenging activity and has no effect on anti-oxidant enzyme expression in HFL1. This difference may be because of the differences in cell type or evaluation method.

Oxidative stress activates the MAPK family, which is a key component of the signaling pathways regulating various intracellular processes, including apoptosis and release of inflammatory cytokines [17]. As previously reported [18], CSE exposure significantly increases the phosphorylation levels of JNK and p38; in contrast, here, NYT only inhibited CSE-induced phosphorylation of JNK but not that of p38. JNKs are classic stress-activated protein kinases that are potently and preferentially activated after various cell stresses, including inflammatory cytokines, and growth factor withdrawal. In the case of apoptosis, JNK is involved in both intrinsic and extrinsic apoptotic pathways [19]. In the intrinsic apoptotic pathway, phosphorylated JNKs stimulate the expression of apoptosis-specific genes through target transcription factors, activate pro-apoptosis factors, such as BAX, and induce mitochondrial outer membrane permeabilization, leading to the release of cytochrome c from the mitochondria [20, 21]. The released cytochrome c forms apoptosomes, which trigger the caspase 9 cascade leading to the cleavage of caspase 3, caspase 6, and PARP, and consequently resulting in cellular apoptosis [22]. In addition, JNK inhibits Bcl-2 activity, which is an anti-apoptotic protein, in both the direct and indirect pathways [23]. In contrast, the extrinsic pathway is characterized by apoptotic signaling initiated by the activation of death receptors by their respective ligands, such as TNF-α. Once the death receptor is activated, adaptor molecules, such as FAS-associated death domain protein, are recruited to the receptor complex, which activates caspase 8 [19]. Activated caspase 8 directly cleaves caspase 3 and caspase 7, leading to apoptosis. JNKs promote the phosphorylation and subsequent cleavage of Bid, leading to the inhibition of anti-apoptotic proteins such as XIAP and cIAP1, which are inhibitors of caspase 8 [24, 25]. Inhibition of XIAP and cIAP1 leads to the activation of caspase 3 and caspase 7, ultimately leading to apoptosis. A previous study has demonstrated that CSE induces apoptosis at lower concentrations (10%–25%) and necrosis at higher concentrations (50%–100%) in HFL1 cells, and that low concentrations of CSE exposure on HFL1 cells induces apoptosis more than it induces necrosis [26, 27]. In the present study, LDH leakage after exposure to 5% CSE was completely inhibited by Z-VAD-FMK treatment, a caspase inhibitor, and not necrostatin-1, a necrosis inhibitor. In addition, NYT treatment inhibited the cleavage of both caspase 3 and PARP at 24 h after CSE exposure. These results suggest that NYT treatment might inhibit CSE-induced activation of intrinsic and/or extrinsic apoptosis pathways through the inhibition of JNK activation.

JNKs are phosphorylated by the upstream dual specificity molecules MAPKK, MKK4, or MKK7; however, NYT did not change the phosphorylation level of MKK4. These results suggest that NYT treatment may activate JNK phosphatase. Among the phosphatases of MAPK, dual specificity phosphatase 16 (DUSP16) is a JNK-specific phosphatase [28, 29]. The forced expression of DUSP16 in COS-7 cells showed suppression of MAPK signal activation, especially that of JNK phosphorylation. In addition, rosiglitazone, a specific ligand of peroxisome proliferator-activated receptor γ (PPARγ), has been reported to upregulate DUSP16 expression and inhibit JNK phosphorylation induced by lipopolysaccharides [30]. In contrast, oleanolic acid, a natural compound present in *Panax ginseng*, has been reported as a potent PPARγ agonist [31]. Therefore, NYT might upregulate DUSP16 expression through PPARγ activation, leading to JNK signal inhibition. However, further investigation is necessary to clarify the detailed mechanism of action of NYT in CSE-induced apoptosis.

The limitations of this study should be acknowledged. The NYT extract powder used in the present study contained numerous compounds that are not absorbed in the body. Therefore, these results might not reflect the actual clinical effects of NYT. To clarify the mechanism of action of NYT in the lungs, it is necessary to evaluate the effect of NYT on cigarette smoke-induced lung injury and apoptosis in mice and humans. Further, we treated cells with NYT for 24 h prior to CSE exposure. We had initially hypothesized that NYT suppressed CSE-induced apoptosis by modulating the expression levels of several proteins, such as that of anti-oxidant enzymes. For this reason, the cells were treated with NYT for 24 h, whereas the cells were treated with other chemicals for a lesser duration before the CSE exposure [32]. However, we demonstrated that NYT might function as a JNK inhibitor; therefore, NYT treatment for a shorter time before CSE exposure might also inhibit CSE-induced apoptosis.

In conclusion, this study clarified that NYT has a protective effect against CSE-induced apoptosis and IL-8 release. In addition, NYT inhibited CSE-induced JNK phosphorylation, but not p38 phosphorylation. These results indicate that NYT possibly functions as a JNK-specific inhibitor, leading to the inhibition of CSE-induced apoptosis and inflammation. Finally, these results suggest that NYT may be a promising therapeutic agent for patients with COPD.

## Supplementary Information

**Additional file 1:** **Supplementary Figure 1.** The full-length blot images for cleaved PARP and caspase 3.

**Additional file 2:** **Supplementary Figure 2.** The full-length blot images for ERK1/2, JNK, p38 and MKK4.

## Data Availability

The datasets used and/or analyzed during the current study available from the corresponding author on reasonable request**.**

## Abbreviations

COPD: Chronic obstructive pulmonary disease
NYT: *Ninjinyoeito*
CSE: Cigarette smoke extract
LDH: Lactate dehydrogenase
CCK8: Cell counting kit-8
ELISA: Enzyme-Linked Immuno Sorbent Assay
IL-8: Interleukin-8
ROS: Reactive oxygen species
