# Supplementary figures and images for: Ninjinyoeito ameliorated cigarette smoke extract-induced apoptosis and inflammation through JNK signaling inhibition in human lung fibroblasts
Source: BMC Complement Med Ther. 2022 Mar 31;22:96. doi: 10.1186/s12906-022-03574-5 (PMC8973640; doi:10.1186/s12906-022-03574-5)

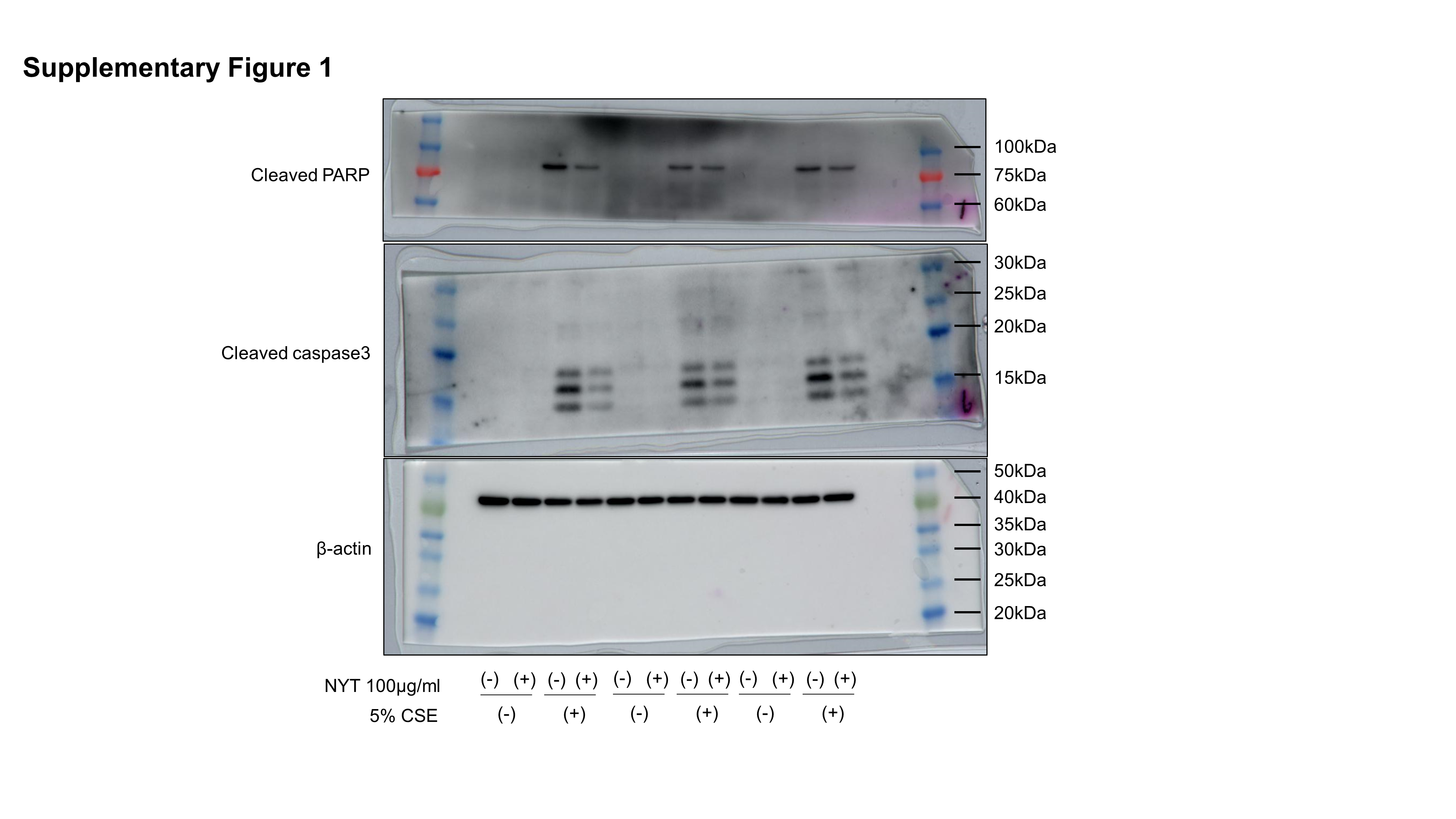

Supplement: Supplementary file 1 — Additional file 1: Supplementary Figure 1. The full-length blot images for cleaved PARP and caspase 3. [file 12906_2022_3574_MOESM1_ESM.tif]

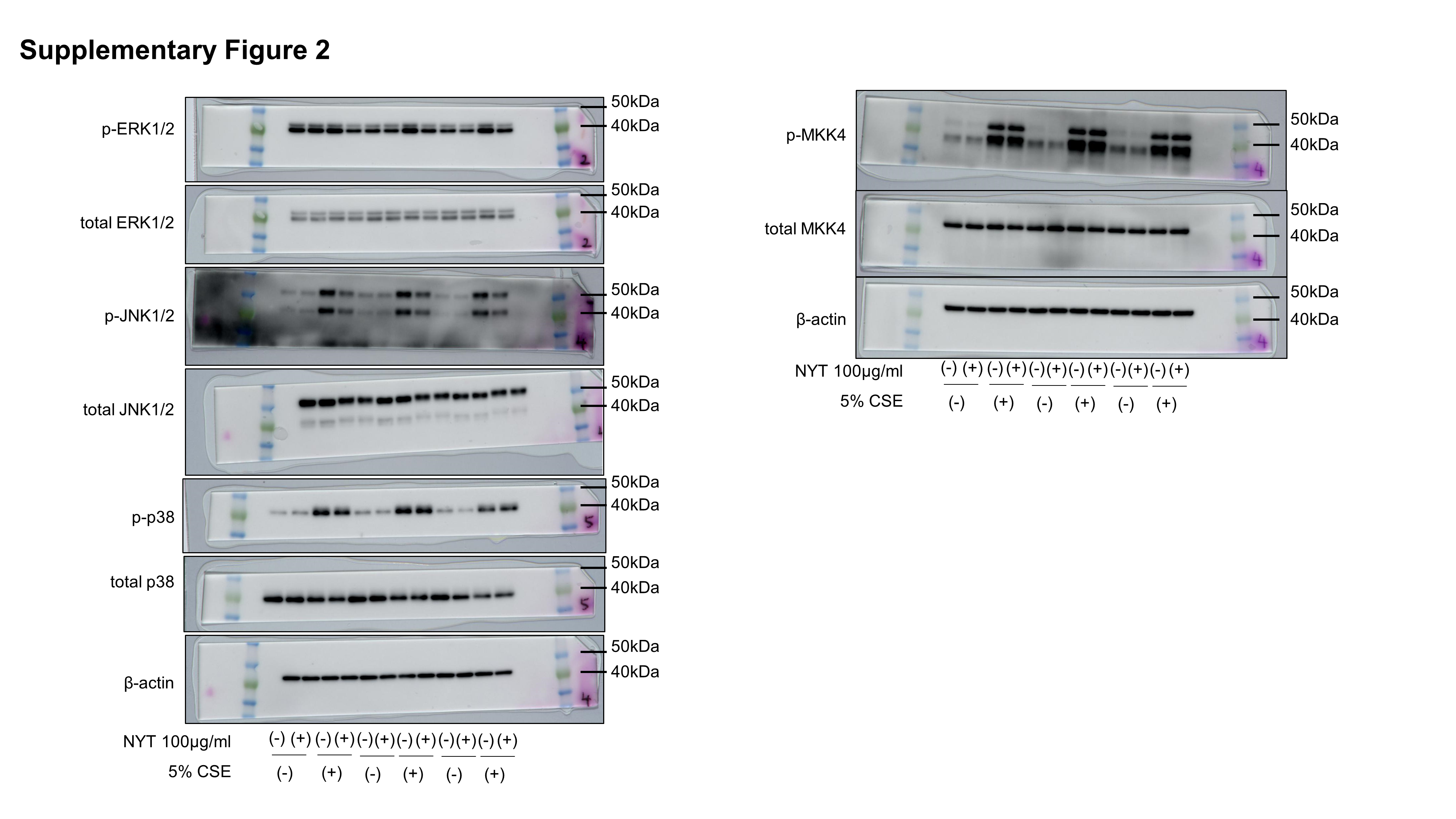

Supplement: Supplementary file 2 — Additional file 2: Supplementary Figure 2. The full-length blot images for ERK1/2, JNK, p38 and MKK4. [file 12906_2022_3574_MOESM2_ESM.tif]
